# A panel of clinical and neuropathological features of cerebrovascular disease through the novel neuroimaging methods

**DOI:** 10.1590/1980-57642016dn11-040003

**Published:** 2017

**Authors:** Gilberto Sousa Alves, Luiza de Amorim de Carvalho, Felipe Kenji Sudo, Lucas Briand, Jerson Laks, Eliasz Engelhardt

**Affiliations:** 1 Departamento de Medicina Interna, Universidade Federal do Ceará, CE, Brazil.; 2 Departamento de Psicologia, Pontifícia Universidade Católica do Rio de Janeiro, RJ, Brazil.; 3 Instituto D'Or de Ensino e Pesquisa, Rio de Janeiro, RJ, Brazil.; 4 Instituto de Psiquiatria, Universidade Federal do Rio de Janeiro, RJ, Brazil.; 5 Programa de Pós-Graduação em Biomedicina Translacional (BIOTRANS), Unigranrio, Duque de Caxias, RJ, Brazil.; 6 Setor de Neurologia Cognitiva e do Comportamento, Instituto de Neurologia Deolindo Couto (INDC-CDA/IPUB), Rio de Janeiro, RJ, Brazil.

**Keywords:** neuroimaging, vascular, PET, MRI, diffusion tensor imaging, DTI, novel methods, neuroimagem, vascular, PET, ressonância magnética, imagem de tensor de difusão, DTI, métodos inovadores

## Abstract

The last decade has witnessed substantial progress in acquiring diagnostic biomarkers for the diagnostic workup of cerebrovascular disease (CVD). Advanced neuroimaging methods not only provide a strategic contribution for the differential diagnosis of vascular dementia (VaD) and vascular cognitive impairment (VCI), but also help elucidate the pathophysiological mechanisms ultimately leading to small vessel disease (SVD) throughout its course.

**Objective:**

In this review, the novel imaging methods, both structural and metabolic, were summarized and their impact on the diagnostic workup of age-related CVD was analysed. Methods: An electronic search between January 2010 and 2017 was carried out on PubMed/MEDLINE, Institute for Scientific Information Web of Knowledge and EMBASE.

**Results:**

The use of full functional multimodality in simultaneous Magnetic Resonance (MR)/Positron emission tomography (PET) may potentially improve the clinical characterization of VCI-VaD; for structural imaging, MRI at 3.0 T enables higher-resolution scanning with greater imaging matrices, thinner slices and more detail on the anatomical structure of vascular lesions.

**Conclusion:**

Although the importance of most of these techniques in the clinical setting has yet to be recognized, there is great expectancy in achieving earlier and more refined therapeutic interventions for the effective management of VCI-VaD.

## RESUMO.

A última década vem testemunhando avanços substanciais na aquisição de marcadores biológicos para o diagnóstico da doença cerebrovascular (DCV). Os métodos de neuroimagem avançados não apenas fornecem uma contribuição estratégica para o diagnóstico diferencial do comprometimento cognitivo vascular (VCI) e demência vascular (VaD), mas contribuem substancialmente na elucidação dos mecanismos fisiopatológicos relacionados à doença de vasos pequenos (SVD) e sua progressão clínica.

**Objetivo:**

Nesta revisão, métodos de imagem estruturais e metabólicos foram descritos e sua importância diagnóstica analisada, particularmente na investigação da CVD relacionada ao envelhecimento. Métodos: uma pesquisa eletrônica de janeiro de 2010 a 2017 foi realizada através do PubMed/MEDLINE, do *Instituto de Informação Científica Web of Knowledge* e da EMBASE.

**Resultados:**

O emprego de estudos de multimodalidade plenamente funcional com Ressonância Magnética (MR)/ Tomografia por Emissão de Pósitrons (PET) representa uma janela para a caracterização clínica mais detalhada da VCI-VaD; com relação à neuroimagem estrutural, a ressonância magnética em 3,0 T vem permitindo varreduras com maior resolução e matrizes de imagem mais elevadas, cortes mais delgados e maior detalhamento anatômico das lesões vasculares.

**Conclusão:**

Embora a importância da maior parte dessas técnicas no cenário clínico aguarde reconhecimento, há uma grande expectativa de que o seu uso favoreça intervenções terapêuticas progressivamente mais precoces e refinadas para o gerenciamento efetivo do VCI-VaD.

## INTRODUCTION

Vascular cognitive impairment (VCI) is an umbrella term denoting a continuum of behavioral and cognitive deficits associated with cerebrovascular disease (CVD).^1^^-^ 3 CVD is estimated to occur in one third of the population, often being recognized as a pathological finding on conventional Magnetic Resonance Imaging (MRI).^1^^,^ 3 ^,^ 4 Depending on the site, intensity, and severity, CVD may either cause or contribute to further cognitive impairment.^2^^,^ 5

Over the last decade, there has been substantial progress in acquiring diagnostic biomarkers for the diagnostic workup of neurodegenerative and vascular disorders.^2^^,^ 6 *In vivo* brain imaging has been applied for several decades to identify brain structural (disease-specific atrophy) and functional (disease-specific hypometabolism) abnormalities. Advanced neuroimaging methods not only provide a strategic contribution for the differential diagnosis of vascular dementia (VaD), but also help elucidate the pathophysiological mechanisms ultimately leading to small vessel disease (SVD) throughout aging.^6^ One example of the growing importance of structural and functional imaging markers on the diagnostic work up of dementia is that the fifth edition of the DSM (2013) has changed to include a broader definition of cognitive impairment, the neurocognitive disorder. These criteria stress the need to support the etiological diagnosis with neuroimaging markers.^6^

In a relatively short period, particularly in the last 15 years, structural neuroimaging has evolved from a quite artisanal approach – focused on the delimitation of pre-defined Region of interest (ROIs) – to powerful volumetric-based morphometry (VBM) analysis,^7^^,^ 8 a measure based on a voxel-wise comparison of highly localized gray matter (GM) regions between two clinical groups. VBM tests for residual tissue concentration differences that remain after spatial normalization into the same standardized stereotaxic space and method calculations rate the within-voxel concentrations of GM (i.e., differences in the proportion of GM contained within a given voxel).^9^^,^ 10 Accordingly, the specific contribution of molecular imaging provided by nuclear medicine techniques such as Single Photon Emission Computed Tomography (SPECT) and Positron Emission Tomography (PET) has been profound, with major improvements regarding specificity, imaging resolution, and more recently, functional multimodality.

Early reports from structural studies have identified subcortical hyperintensities as macroscopic white matter (WM) changes which have been cited by several reports as associated with CVD-VCI, mood disorders, executive dysfunction and higher conversion to dementia. In functional terms, it is hypothesized that cognitive deficits observed in subcortical VCI arise when infarcts in the WM lead to the disruption of neuronal circuits connecting cortical and subcortical structures. Despite the substantial progress on the characterization of cognitive deficits and the early identification of minor vascular lesions, other important issues related to CVD, such as the neuropathological etiology of these lesions, remain a subject of intensive research in the last decade, with studies evolving to address the relationship between normal-appearing WM and amyloid angiopathy or Wallerian Degeneration.^11^^-^ 13 On the other hand, cerebral microbleeds (CMB) or cerebral microhemorrhages (CMH) are small hypointense lesions with variable cut-off size – typically between 5 and 10 mm – that have been attracting growing interest in recent years. Previous literature has shown an increased number of CMBs in MCI (around 11%) and there is an extensive debate on the significance of these lesions in terms of higher conversion to dementia.^14^

In this brief review, we aim to summarize some of these novel implementations, both in the macro and microanatomy and radiotracer aspects, and discuss their impact on the diagnostic workup of age-related cognitive disorders, focusing on the field of VCI-VaD.

## METHODS

A review of the literature (Table 1) published between January 2010 and 2017 was performed through searches on the electronic databases PubMed/MEDLINE (http://www.ncbi.nlm.nih.gov/pubmed/), Institute for Scientific Information Web of Knowledge (http://www.isiknowledge.com) and EMBASE (http://www.embase.com), using the following terms: “structural neuroimaging”, “cerebrovascular”, “vascular dementia”, “vascular cognitive impairment” “aging”, “diffusion tensor imaging”, “DTI”, “MRI”, “VBM”, “molecular neuroimaging”, “SPECT” and “PET” search. Firstly, the complete abstract was read, with the first paper selection. A second selection included the full reading of the papers. Articles were included if they focused on clinical and therapeutic applications of novel neuroimaging techniques in the assessment of cognitive symptoms of VCI-CVD-VaD. Although we designed a non-systematic review, article retrieval and selection were performed following the main recommendations of the Moose guidelines.^15^

**Table 1:** Structural and metabolic imaging studies with VCI and VaD.

Authors (sample)	Subjects (Mean age/Gender M:F)					Methods	Neuroimaging method	Neuroimaging vs behavioral changes
Lanna et al. 2012^104^ (n=6)	Patients (n=6) 61.3/2:4					Clinical/neuropsychological and brain MRI/SPECT examination.	1.5T MRI; SPECT with IV line 740MBq of (99 m) Tc.	Moderate to severe dementia associated with strategic strokes as a result of neuronal disruption in multimodal areas.
Holst et al. 2012^105^ (n=440)	SVD no dementia (n=440) 65.2/239:201					Clinical/ neuropsychological and brain MRI/DTI examination.	1.5T MRI; DTI processing.	Microstructural integrity of the cingulum is specifically related to episodic memory function, notably verbal memory, in non-demented elderly with SVD. Cingulum integrity is significantly lower in participants with greater hippocampal alteration than those with hippocampal integrity.
Hilal et al. 2016^106^ (n=424)	NCI (n=96) 68.4/44:52	CIND (n=177) 71.5/91:86 CIND (n=107) VCIND (n=70)	Dementia (n=151) 76.1/57:94 Alzheimer (n=121) VaD (n=30)			Clinical/neuropsychological MRI/MRA examination.	3.0T MRI; 3D TOF MRA.	ICS was shown to be independently associated with VCIND, whereas in dementia, this association was mediated by cerebral ischemic damage, independently of cardiovascular risk factors. This further suggests that ICS is a marker of cerebral or generalized atherosclerosis and may be a viable treatment target.
Lawrence et al. 2013^62^ (n=165)	SVD (n=115) 70.0/78:37	Control (n=50) 70.3/35:22				Clinical/neuropsychological and brain MRI/DTI examination.	1.5T MRI; DTI processing.	Patients with symptomatic SVD and radiological leukoaraiosis exhibited greater impaired performance in executive function, processing speed and working memory than in episodic memory. Executive memory and processing speed were associated with lacunar infarct load, reduced brain volume, and microstructural WM alterations (DTI)
Liu et al. 2014^65^ (n=69)	SIVD (n=34) 69.0/21:13	Control (n=35) 68.0/22:13				Clinical/neuropsychological and brain MRI examination.	3T MRI 1.0 mm.	Cortical thinning of the hippocampus and volume reduction of the caudate nucleus was associated with poorer global cognitive performance in SIVD patients. Cortical thinning and cognitive decline were not related to severity of WML. The automated measures of deep gray matter also revealed significant volume reductions in the amygdala and nucleus accumbens.
Cavallari et al. 2014^107^ (n=56)	Subjects (n=56) 82/24:32					Clinical/neuropsychological and brain MRI/DTI examination.	3T MRI; DTI processing.	The observed association between DTI changes in the thalamus at baseline and the rate of WMH accrual singles out thalamic FA as a promising candidate surrogate marker of cerebral SVD progression, and points to a possible causal role of thalamic damage in the accrual of WMHs.
Guerra et al. 2014^108^ (n=25)	SIVD (n=7) 78.1/02:05	Control (n=9) 75.0/03:06	AD (n=9) 74.3/00:09			Clinical/neuropsychological TMS mapping.	TMS mapping.	SIVD patients showed higher degree of WMH and lesser extent of medial temporal atrophy than AD patients
Jeong et al. 2014^109^ (n=587)	END (n=79) 64.4/44:35	No END (n=508) 65.5/303:205				Clinical/neuropsychological and brain MRI examination.	1.5T or 3T MRI.	SSSIs who exhibited relevant artery stenosis and branch atheromatous lesions were more likely to develop END but the association was not documented in the SSSI patients with WMH, old lacunar infarctions, or CMBs.
Garriga et al. 2015^110^ (n=105)	MCI (n=50) 72.0/26:24	VaD (n=6) 73.0/03:03	AD (n=31) 76.0/12:19	LBD (n=6) 73.0/03:03	PDD (n=12) 77.0/10:02	Clinical/neuropsychological MRI or CT and ^123^I-FP-CIT SPECT examination.	1.5T MRI; CT and ^123^I-FP-CIT SPECT.	^123^I-FP-CIT SPECT is a useful tool to discriminate MCI from overall dementia subtypes. However, ^123^I-FP-CIT SPECT could not discriminate among different dementia types.
Lin et al. 2015^111^ (n=50)	VCIND (n=22) 72.8/14:08	Control (n=28) 70.9/16:12				Clinical/neuropsychological and brain DTI examination.	3.0T MR DTI processing.	SIVD patients displayed abnormal WM connectivity in all supratentorial regions, involving projection, association, and commissural fibers; the severity of damage in WM tracts was correlated with cognitive dysfunction in SIVD patients.
Ostojic et al. 2015^112^ (n=50)	VD (n=25) 69.5/16:09	Control (n=25) 71.6/15:10				Clinical/neuropsychological and brain MRI/DTI examination.	3T MRI; DTI processing.	No significant group differences for FA were found in the left and right hippocampus; no significant differences between hemispheres in either HC and VD group were reported.
Wu et al. 2015^113^ (n=60)	SIVD (n=30) 65.3/17:13	Control (n=30) 63.9/16:14				Clinical/neuropsychological and brain MRI/DTI examination.	1.5T MRI; DTI processing.	SIVD patients had significantly reduced corpus callosum size compared with healthy controls. Patients with SIVD showed significantly lower FA values in the genu and splenium of the corpus callosum as compared with healthy controls.
Baykara et al. 2016^89^ (n=710)	CADASIL exploratory (n=113) 49.1/43:61	CADASIL validation (n=57) 53.4/38:19	Sporadic SVD (n=444) 65.3/243:201	Memory clinic patients with SVD (n=105) 74.9/54:51		Clinical/neuropsychological and brain MRI/DTI/PSMD examination.	MRI; DTI with PSMD processing.	The study established a novel-imaging marker (PSMD) for SVD. PSMD combined the analysis of DTI-WM tract skeletonization and MD histogram; PSMD was associated with SVD pathology but not with neurodegenerative pathology. An important association between PSMD and processing speed deficits across all study samples was reported.
Veluw et al. 2016^59^ (n=5)	Cases with CAA (n=5) 85.0/03:02					Clinical/neuropsychological and brain histopathological and in vivo and ex vivo MRI examination.	Ex vivo 7.0T MRI.	CMBs and microinfarcts appeared to be the most frequent marker of focal bleeding and focal ischaemic injury in SVD (particularly CAA), and therefore are important candidate biomarkers for clinical trials.
Thong et al. 2014^87^ (n=100)	Control (n=25) 68.00/11:14	Mild VCIND (n=25) 66.20/19:06	MSVCI (n=30) 69.87/14:16	AD (n=20) 74.25/06:14		Clinical/neuropsychological and brain MRI/HARDI examination.	3T MRI 1 mm and HARDI 3 mm.	Compared to the Mild VCIND group, MSVCI subjects showed thinner cortex in the left superior frontal gyrus, cingulate, temporal pole, and middle temporal gyrus; in contrast, no statistical differences between groups in cortical thickness in the right hemisphere were reported.
Mascalchi et al. 2016^114^ (n=36)	CADASIL (n=18) 42.9/07:11	Control (n=18) 41.2/10:8				Clinical/neuropsychological and brain MRI/DTI examination.	3T MRI ; 1mm ; DTI processing; TBSS.	CADASIL patients showed extensive almost symmet­ric areas of significantly increased radial and mean diffusivities and of significantly decreased axial dif­fusivity and FA that involved the cerebral WM, the thalami, and the corpus callosum.
Pasi et al. 2017^115^ (n=36)	CAA-ICH (n=191) 74.9/95:96	HTN-ICH (n=125) 66.1/73:52				Clinical/neuropsychological and brain MRI examination.	1.5T MRI.	The topographic distribution of lacunes (lobar vs deep) helped distinguish the underlying SVD subtype (CAA vs HTN-SVD) in patients with primary ICH, regardless of age status, diagnosis of hypertension or other MRI markers of SVD severity. Lobar lacunes seemed to have a closer relationship with WMH, suggesting a possible common origin.

AD: Alzheimer's Disease; MRI: Magnetic Resonance Imaging; DTI: Diffusion Tensor Imaging; TBSS: Tract-Based Spatial Statistics; TMS: Transcranial Magnetic Stimulation; FA: Fractional Anisotropy; WM: White Matter; WMH: White Matter Hyperintensities; CC: corpus callosum; SPECT: Single Photon Emission Computed Tomography; IV: intravenous; VCIND: Vascular Cognitive Impairment, No Dementia; CIND: Cognitive Impairment, No Dementia; NCI: No Cognitive Impairment; 3D TOF MRA: Three-dimensional Time-of-flight Magnetic Resonance Angiography Images; END: Early Neurological Deterioration; SIVD: Subcortical Ischaemic Vascular Dementia; SSSIs: Single Small Subcortical Infarctions; SVD: Small Vessel Disease; PDD: Parkinson Disease With Dementia; LBD: Lewy Body Dementia; ICH: Intracerebral Hemorrhage; HTN-SVD: Hypertensive Small Vessel Disease; PSMD: Peak width of skeletonized mean diffusivity; HARDI: High Angular Resolution Diffusion Imaging;

* gender not discriminated,

** age not discriminated,

** Mean Age and gender were not described, but no significant differences were found between groups regarding these parameters.

## RESULTS

A total of 790 articles were retrieved and 127 remained for further analysis, after primary exclusion. A total of 25 studies were subsequently considered eligible for inclusion and discussion.

PET and SPECT. Both PET and SPECT have been intensively applied in the last 20 years to quantify changes in regional brain function induced by age-related disorders^16^^,^ 17 The most widely utilized PET tracer in cognitive disorders is 2-[18F] fluoro-2-Deoxy-D-glucose (FDG) PET for measurements of cerebral metabolic rate of glucose (CMRglc), an indicator of different parameters, e.g., neuronal activity, oxygen consumption, synaptic alterations and molecular changes. 16 ^,^ 17 Studies have shown that CMRglc reductions occur in AD-risk states,^14^ in preclinical AD,^18^ and correlate with disease progression^19^ with higher accuracy than the Mini-Mental State Examination and ADAS-cog.^20^

Considerable technical improvements have been made in SPECT and PET methodology, propelling the introduction of modern hybrid technology,^21^ which has improved structural – functional assignments that have paved the way for SPECT/CT and PET/CT towards accepted clinical imaging standards.^16^^,^ 22 The beneficial effects of hybrid systems are clear for brain scans, since post-hoc software-based image fusion of independently acquired imaging data may be employed as a well-established and precise method.^23^ Advanced technology implemented in modern hybrid devices offers the opportunity to reduce image acquisition times,^22^ and to use low-dose CT for accurate attenuation correction of brain scans on SPECT/CT and PET/CT. More recently, with the advent of simultaneous MR/PET,^24^ novel solutions for adequate attenuation correction have been proposed,^25^^-^ 27 but also improved algorithms for head motion correction utilizing MRI-based motion tracking in combination with PET list mode data motion correction,^28^^,^ 29 having a major impact on the spatial resolution of brain imaging studies.

### True multimodality.

The most fundamental advantage of MR/PET is a major advancement in true multimodality,^21^^,^ 30 e.g. structural-functional and functional-functional. Due to the distinctiveness of structural MRI sequences yielding quantitative MR applications,^31^^,^ 32 a more refined clinical structural depiction of brain lesions has become possible, along with the functional characterization provided by PET tracer measurements.

Indeed, all the methodological improvements of recent years have also led to important enhancement in the diagnostic characterization of cognitive disorders, improving the specificity and sensitivity of human imaging biomarker studies.^19^ For instance, the possibility of full functional-functional multimodality in simultaneous MR/PET; hybrid protocols offer for instance parallel FDG PET and MRI-spectroscopic imaging,^24^ improving the molecular imaging characterization of CVD and neurodegenerative disorders; or, parallel dynamic ligand acquisitions in combination with functional MRI techniques (e.g. BOLD fMRI, resting state fMRI, continuous arterial spin labeling, contrast-enhanced perfusion techniques, etc.) under pharmacological or non-pharmacological experimental challenges, are expected to advance molecular characterization of neurodegenerative disorders and CVD in clinical human neurosciences. Finally, improvements in PET imaging temporal resolution have opened a field for integrating time-of-flight^33^^,^ 34 information into the reconstruction process, leading to the high resolution of today's PET/CT systems, again, improving imaging capabilities, potentially reducing acquisition times and improving the specificity of the imaging set-up.

While in AD the best-recognized biomarkers that can be detected in cerebrospinal fluid and blood are amyloid-b, tau-protein and phosphorylated tau-protein (phospho-tau), for VaD no specific biomarker is available. Nevertheless, in VaD, FDG PET usually differentiates widespread areas of focal cortical and subcortical hypometabolism from the pattern typically found in AD, with markedly lower metabolic rates in temporal-parietal lobes.^17^ Additionally, in VCI-VaD, the metabolic ratio seems to be generally higher than in the AD group.^17^ Another study conducted by Kim et al.^35^ investigated the profile of negative [(subcortical vascular dementia)(n=24)] and positive [(AD), (n=81)] amyloid-b patients using 11C PiB PET. When compared to AD, the negative amyloid-b (subcortical vascular patients) cases showed more pronounced cortical thinning in the bilateral inferior frontal, superior temporal gyri and orbitofrontal lobes. Findings also evidenced that, in these areas, SVD independently contributed to cortical atrophy through different mechanisms than those of AD.^35^

More recently, F-18 labeled amyloid PET tracers have been introduced, including compounds such as [18F]Florbetaben,^36^^-^ 38 [18F]Flutemetamol^39^^,^ 40 and [18F]Florbetapir.^41^^,^ 42 These novel compounds offer the major advantages of a longer half-life of the radioactive label (110 min), allowing a much wider distribution of amyloid PET scans, even in institutions without an on-line cyclotron facility. Another innovative method to specifically characterize AD pathology by PET imaging is through the use of selective in-vivo tau PET tracers, which allow the quantifying of tau aggregation in the brain.^43^^,^ 44 Neuropathological studies of AD show a strong association between tau deposits, decreased cognitive function, and neurodegenerative changes, and selective tau imaging enables these associations to be explored *in vivo*.^43^

Although qualitative findings achieved with amyloid tracers are robust, quantitative measures of amyloid tracer retention show considerable variability across centers. Therefore, standardization of acquisition protocols, subject management, tracer administration, image quality control, and image processing and analysis methods has become an important issue for improving the accuracy of quantitative amyloid PET measurements.^45^ This is of particular importance for longitudinal multi-center studies, and for improving the sensitivity of intervention effects targeting amyloid clearance.^46^ Recently, a novel method for standardization denominated ‘Centiloids' was introduced, which attempts to standardize the quantitative amyloid data by relating “nonstandard” analysis methods to a ‘standard' PIB PET data analysis and expressing the data after transformation into the so-called ‘Centiloid scale'.^45^

Quantitative Susceptibility Mapping (QSM) of the Brain. Many biological processes, including regulation of protein expression,^47^^,^ 48 oxygen transport and neurotransmission require the presence of iron. The accumulation of iron may be found throughout normal aging,^49^^,^ 50 for instance in the basal ganglia and hippocampus and also in subcortical regions.^49^ In a variety of cognitive related disorders, including CVD, iron accumulation is thought to play an important role, possibly due to biochemical alterations related to neurodegeneration (e.g., oxidative stress, abnormal neuronal connectivity), although the exact mechanisms are not fully understood. In rodent models, iron deposition was associated with WM disruption and atrophy by inducing endothelial cell damage.^50^^,^ 51 More recently, improved quality and accuracy in the quantification of iron by MRI has been achieved through quantitative susceptibility mapping (QSM). In the field of vascular-related disorders, despite limited data, there is increasing evidence pointing to iron accumulation in putamen and caudate nucleus when compared to healthy controls.^50^^,^ 52 Similar findings were also reported with CADASIL (cerebral autosomal-dominant arteriopathy with subcortical infarcts and leukoencephalopathy), a genetically defined form of early SVD. 52 ^,^ 53 Although most of the literature reported a predominant pattern of iron deposition in subcortical areas, accumulation in greater vessels has also been mentioned, in this case leading to more extensive vascular damage.^50^ Taken together, these support the idea that iron accumulation is a marker of neurodegeneration and endothelial damage, regardless of the underlying process.

High field MRI of the Brain. The main advantage of 3.0 T over lower-field MR scanners is a better sign-to-noise ratio (SNR), which increases roughly linearly with the strength of the magnetic field.^54^ From 2004 on, a number of studies have been conducted to assess white matter damage in diseases such as multiple sclerosis,^32^ Alzheimer's disease (AD),^55^ and adrenoleukodystrophy^19^ with 3.0 or 4.0 T MR scanners, and evaluation of iron deposition in neurodegenerative and cerebrovascular disease at 7 T.^49^^,^ 54 Consequently, imaging at 3.0 T enables higher-resolution scanning with larger imaging matrices, thinner slices and more detail on anatomical structure, without extending (or extending minimally) the scan acquisition time. These advantages come with a trade-off of increased sensitivity to field inhomogeneity (deviation of the local magnetic field from its average value) and changes in relaxation times, in turn producing changes in image contrast.^54^ At comparable acquisition times, images obtained at 3.0 T have a higher quality with an improved resolution over images obtained at 1.5 T. Alternatively, 3.0 T MRI can be used to obtain acceptable images, similar to those obtained at 1.5 T, but at a fraction of the time, thus reducing potential motion artifacts and providing greater comfort for patients.

Regarding the study of vascular-related pathology, a major development is underway, particularly with the advent of 7 T MRI; previous attempts have been made to distinguish between vascular occlusion and microinfarction versus demyelinating disease, for instance in the differential diagnosis at 7 T between Susac syndrome and Multiple Sclerosis.^56^^,^ 57 Accordingly, the anatomic modifications involving dilated perivascular spaces have been investigated and quantified with greater accuracy using 7 T MRI,^58^ showing different patterns and quantification, for instance, in stroke, migraine, CADASIL, dementia, AD, and mild cognitive impairment. Furthermore, 7 T studies may be more successful in providing a more detailed picture of the neuropathology of closely related conditions, such as the case of cerebral microinfarcts, whose regional distribution (intracortical and juxtacortical location)^59^ may indicate chronic or acute lesions, with the latter being described as gliotic cerebral microinfarcts with hemorrhagic components. These findings can be confidently extended by *in vivo* MRI in the context of aging and dementia.

Another useful tool for analyzing subcortical vascular lesions is to mask white matter hyperintensities (WHM). Some of the methods are based on the Expectation-Maximization (EM) algorithm,^60^ which differentiates brain tissue into WM, GM and cerebrospinal fluid (CSF). In addition, tissue segmentation from T1 and Flair images through the EM algorithm also enables the segmentation of lacunar infarcts.^61^ Finally, the combination of high-field MRI techniques and novel image sequences, particularly susceptibility-weighted imaging (SWI), has improved the detection of CMB.^14^

Cortical thinning and cortical surface analysis. Hippocampal and GM reductions are often described in different forms of CVD and may indicate, with greater likelihood, conversion to dementia.^7^ In subjects with subcortical ischaemic vascular dementia (SIVD), several studies have observed decreased gray matter both in the total volume^62^ or in regional territories,^7^^,^ 63 for instance the frontal and temporal lobes. In addition, hippocampal volume atrophy, particularly in the CA1 subfield, seems to be vulnerable to vascular-related events, for instance, hypoxia and ischemia, as suggested by rodent-model^64^ and structural neuroimaging^65^ investigations. More recently, it has been shown that cortical volume, surface area and cortical thickness are closely related, and a reduction in cortical volume may affect either thickness or surface area (or both).^65^ Initial studies have attempted to investigate cortical thinning and found reduction in the perisylvian, medial frontal area and posterior cingulate.^35^^,^ 66 More recently, surface area in gray matter has been found to be reduced in SIVD, particularly in the left temporal lobe and dorsolateral PFC.^65^ Possibly, cortical thinning and atrophy play a greater role in cognitive decline than the occurrence of WML.^67^ Taken together, these findings provide further support for the close relationship between vascular and neurodegeneration, highlighting the anatomical relevance of the perisylvian area as a highly sensitive territory to the effects of cortical thinning, possibly due to ischemic damage of lateral cholinergic pathways and disruption of fibers connecting the nucleus basalis of Meynert to frontoparietal and temporal areas.^68^

The use of support vector machines in CVD. The last decade has witnessed a great deal of effort in the development of methods to quantitatively assess specific CVD markers and devise metrics allowing the quantification of total CVD burden.^69^ Regarding data analysis, machine learning-based algorithms for dementia classification according to the expression of AD-typical metabolic patterns (but also other imaging parameters) are recent developments and have been influencing the field of cerebrovascular disorders. The most employed algorithms include K-means clustering, artificial neural network, random forest and support vector machine (SVM); the potential advantage of SVM is the classification of more than one biomarker combined, further improving performance accuracy, as demonstrated by studies matching FDG-PET and structural MRI, which yielded higher accuracy rates compared to single modality classification.^70^^,^ 71

Despite the enthusiasm and great potential of these methods, problems in defining threshold and clustering approach and the statistical inference for SVM and the use of permutation tests that ignore SVM margin still limit the wide applicability of these methods. Furthermore, a number of drawbacks remain regarding the limited evaluation of findings, particularly the clinical interpretation of disease mechanisms according to the classifier's decision.^69^ Possibly in the future, a better quantification of the total brain burden of CVD through machine learning-based algorithms will help promote both the stratification of patients (rather than using individual features) and understanding of the cause-effect relationship between events that ultimately leads to CVD. Hence, the ambitious achievements of advanced methods of CVD open a large avenue to unravel the core neuropathological mechanisms (e.g., axonal degeneration, myelin breakdown) related to vascular disease.^6^ In the clinical scenario, there is great hope that, in the years to come, SVM can be employed to identify and reverse vascular tissue disease at earlier stages, before lesions become apparent.^69^

**Figure 1:**
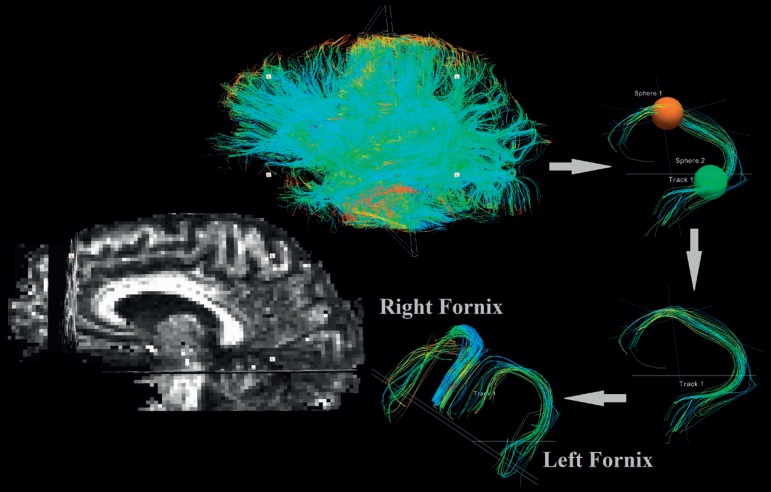
Depiction of virtual delineation by deterministic tractography (Trackvis protocol for tract delineation). Fornix fibers (left and right) are visualized as blue fibers. Figure originally designed by the authors of the manuscript.

Diffusion tensor imaging (DTI) and tractography. Diffusion tensor imaging (DTI) is a variant of MRI that non-invasively measures the diffusion of water *in vivo* brain tissues,^72^^-^ 74 that is highly sensitive for evaluating the microstructure of WM, including the study of axonal organization, density of the fibers and even the integrity of the myelin sheath.^72^^,^ 75 One of the most common proxies of DTI is fractional anisotropy or anisotropy fraction (FA), an indirect measure of the molecule direction in a given set of fibers and bundles of a brain structure.^72^^,^ 73 ^,^ 76 An increasing body of evidence has demonstrated an association of WM microstructural abnormalities and FA decreases in the deep WM and corpus callosum of patients with VCI compared to healthy controls.^9^ Although largely unknown and somewhat speculative, a number of studies have also linked low FA measurements to reduced WM density, loss of axonal coherence of axonal fibers (loss of structural organization), and changes in membrane water permeability.^77^ Another promising approach of DTI in a variety of neuropsychiatric disorders^78^^-^ 83 is the use of tractography, which allows non-invasive three-dimensional identification of fiber tracts^74^^,^ 84 and enables WM bundle reconstruction typically found in post mortem analysis.^85^ Tractography is based on fiber connectivity probability and anisotropic water movement in a specific group of fibers and their surroundings. The technique can be either global or local, probabilistic or deterministic^86^ (Figure 1).

The use of the isotropic HARDI technique, including single shot sequences with no interslice gaps and use of the HARDI atlas, seems to provide more powerful computational analysis and higher precision anatomical examination of WM integrity.^87^^,^ 88 For instance, moderate to severe VCI exhibited increased mean diffusivity (MD) in the temporal lobe and decreased FA in the corpus callosum, superior longitudinal fasciculus (SLF), internal capsule (IC), corona radiate (CR), thalamus and uncinate fasciculus (UNC).^87^ Interestingly, no differences in MD or FA were found between AD and VCI and this may point to distinct trajectories of fiber bundles compromised by these two conditions. In the neuroprogression of AD, for instance, while the posterior U-fibers of the superior longitudinal fasciculus are often compromised, in VCI the most common pattern includes the involvement of neocortical anterior bundles, typically long association fibers, such as the UNC and IC/CR.^87^

Another interesting technique is the peak width of skeletonized mean diffusivity (PSMD), which is based on the analysis of fiber tracts and on the difference between the 95th and the 5th percentiles of the voxel-based MD values within the WM skeleton.^89^ Increasing evidence has shown that the PSMD may substantially reduce contamination from CSF and other spurious structures and enhance the sensitivity of SVD total burden measures.^69^ Recent evidence also indicates greater sensitivity of the PSMD for rating the progression of injury from SVD than the individual volumetric measures of WMH, lacunes and brain total volume. In a cross-sectional investigation of 69 patients with CADASIL, processing speed emerged as the most prominent cognitive domain affected.^89^ The use of STRIVE^89^^,^ 90 criteria on T2-weighted gradient echo images has been employed for the identification of CMB. Conversely, lacunar volumes could be rated by placing a seed-growing algorithm where a seed voxel is placed into a lacune on the 3D-T1 image.^89^

Magnetization transfer (MT). Magnetization transfer (MT) imaging, or magnetization transfer contrast (MTC) magnetic resonance imaging, is a modality of MRI technique based upon the exchange between bonded water in the brain tissue and proton magnetization in free water to characterize brain tissue properties quantitatively.^91^^,^ 92 The magnetization transfer ratio (MTR), derived from MT imaging, has been explored and used to evaluate brain injury in different brain diseases, e.g., multiple sclerosis, Alzheimer's disease, stroke, and epilepsy. In the field of vascular disease, although early reports have been available since 1999, only a small number of studies have employed this approach. One of these investigations included 56 subjects with WML and showed that periventricular WM had lower MTR than deep WM.^93^ Such findings were replicated by later studies showing an association of cognitive impairment with either larger periventricular WM^94^^-^ 96 or reduced MTR in normal appearing white matter (NAWM).^97^ Overall, these studies also support previous evidence showing reduced periventricular MTR in subjects with Binswanger disease (whose cognitive impairment is associated with a proportion of subcortical WML over 25%) compared to non-demented subjects with similar severity of WML, and seem to confirm the sensitivity of MTR for detecting clinically relevant CVD.

## CONCLUSIONS

This review briefly summarizes some of the most promising neuroimaging techniques addressing brain structural and metabolic changes in CVD. Indeed, much has been achieved in terms of unraveling the neuropathological underpinnings and clinical correlates of VCI and VaD; a number of controversial issues, however, still remain. While on the one hand, neuroimaging has evolved quickly in the development of powerful and sensitive methods for studying *in vivo* brain architecture in CVD and related cognitive disorders, most structural techniques are limited by a number of pitfalls. The source of criticism centers on multiple aspects, including the large variability in imaging modalities and procedures (e.g., threshold values for cluster definition), the limited accuracy of DTI-MRI (poor identification of crossing fibers, poor specificity of findings) and low replication of results.^98^^,^ 99

Despite the aforementioned limitations, novel neuroimaging methods offer an enthusiastic debate on the interplay between aging, neurodegeneration and vascular disease; one interesting topic involves, for instance, the pattern of cortical thinning exhibited by CVD and other related cognitive disorders. Indeed, a pattern of cortical thinning in frontal and subcortical areas seem to be closely related to SIVD, contrasting with the temporo-parietal and medial temporal findings typically observed in AD.^35^^,^ 100 Another point of controversy is based on the nature of cortical changes observed in CVD. While much has been discussed on the complex interaction between neurodegeneration and vascular disease, current evidence also suggests that CVD may independently lead to cortical atrophy.^35^ WMH may possibly cause subcortical axonal damage and neuronal disruption of cortical pathways, leading to secondary neuronal body damage and ultimately gray matter atrophy.^35^^,^ 101 Contrasting with the hypothesis of vascular-induced cortical atrophy is the Wallerian degeneration model, which basically conceives WM atrophy as a product of gray matter progressive reduction.^11^^,^ 99 ^,^ 102 ^,^ 103

Thus, the continuous development of brain imaging techniques through new metabolic tracers, molecular compounds, multimodal approaches, microstructural anatomy, disease classifying algorithms and higher field MRI offer an exciting perspective towards a broad comprehension of CVD pathophysiology. Although the importance of most of these techniques in the clinical setting has yet to be recognized, there is great expectancy in achieving earlier and more refined therapeutic interventions for the effective management of dementia.
